# Distinct redox state regulation in the seedling performance of Norway maple and sycamore

**DOI:** 10.1007/s10265-022-01419-3

**Published:** 2022-11-17

**Authors:** Shirin Alipour, Natalia Wojciechowska, Barbara Bujarska-Borkowska, Ewa Marzena Kalemba

**Affiliations:** 1 Institute of Dendrology, Polish Academy of Sciences, ul. Parkowa 5, 62035 Kórnik, Poland; 2 Department of General Botany, Institute of Experimental Biology, Faculty of Biology, Adam Mickiewicz University, Uniwersytetu Poznańskiego 6, Poznań, Poland

**Keywords:** *Acer platanoides*, *Acer pseudoplatanus*, Ascorbate, Glutathione, Nicotinamide adenine dinucleotide (phosphate), Plant growth

## Abstract

Norway maple and sycamore, two *Acer* genus species, have an important ecological value and different sensitivity to stressing factors being currently aggravated by climate change. Seedling growth is postulated to be the main barrier for successful plant establishment under the climate change scenarios. Therefore, the differences in redox regulation during the seedling performance of Norway maple and sycamore were investigated. Seeds of the two *Acer* species exhibited an identical high germination capacity, whereas seedling emergence was higher in sycamores. PCA analyses revealed that there is more diversification in the leaf characteristics than roots. Norway maple displayed a higher chlorophyll content index (CCI) with a similar leaf mass whereas sycamore seedlings exhibited a higher normalized difference vegetation index (NDVI), higher water content, higher root biomass and higher shoot height. Based on NDVI, sycamore seedlings appeared as very healthy plants, whereas Norway maple seedlings displayed a moderate healthy phenotype. Therefore, redox basis of seedling performance was investigated. The total pool of glutathione was four times higher in sycamore leaves than in Norway maple leaves and was reflected in highly reduced half-cell reduction potential of glutathione. Sycamore leaves contained more ascorbate because the content of its reduced form (AsA) was twice as high as in Norway maple. Therefore, the AsA/DHA ratio was balanced in sycamore leaves, reaching 1, and was halved in Norway maple leaves. Nicotinamide adenine dinucleotide phosphate content was twice as high in sycamore leaves than in Norway maples; however, its reduced form (NADPH) was predominant in Norway maple seedlings. Norway maple leaves exhibited the highest anabolic and catabolic redox charge. The higher reduction capacity and the activity of NADPH-dependent reductases in Norway maple leaves possibly resulted in higher CCI, whereas the larger root system contributed to higher NDVI in sycamore. The different methods of controlling redox parameters in *Acer* seedlings grown at controlled conditions provided here can be useful in understanding how tree species can cope with a changing environment in the future.

**Supplementary Information:**

The online version contains supplementary material available at 10.1007/s10265-022-01419-3.

## Introduction

Seedling performance is an attribute which concerns to the early stages of the tree life cycle following germination and is very susceptible to environmental cues, which are predominantly temperature and the availability of soil moisture (Walck et al. 2011) as well as herbivory pressure (Barton and Hanley 2013). Successful seedling establishment ensures the continuity of forest ecosystems, however climate change scenarios evoke worries about the future of ecosystems and biodiversity conservation (Mondoni et al. 2012). Climate change seems to be the most detrimental to seedling emergence stage of the plant life cycle (Walck et al. 2011). A meta-analysis integrating plant responses to climate change revealed inconsistent effects of climate change impacts (projected warming, early snowmelt, changes in precipitation, nutrient availability) on seedling establishment (Vázquez-Ramírez and Venn 2021), indicating that species-specific, provenance-specific (Prober et al. 2015), and ecotype-specific (Curasi et al. 2019) responses to the climate change scenario exists, which are driven by local environmental conditions (Hansen and Turner 2019). The neutral response of seedlings to decreases in precipitation and warmer temperatures is optimistic only for high-altitude and high-latitude regions (Vázquez-Ramírez and Venn 2021). In other regions, global climate change is suspected to delay, enhance or completely inhibit seedling establishment. Therefore, knowledge about seedling development at optimal conditions of each tree species is needed to understand future response strategies to a changing environment.

Norway maple (*Acer platanoides* L.) and sycamore (*Acer pseudoplatanus* L.) are native, widespread, and fast-growing species in Europe (Suszka et al. 1996). Norway maple is a secondary species of temperate mixed forests, whereas sycamore dominates mixed softwood deciduous forests (Caudullo and de Rigo 2016; Pasta et al. 2016). Both Norway maple and sycamore are used as ornamental and shade trees for urban planting. In particular, Norway maple is known as an invasive species, termed urban tree invader, that outcompetes native trees in North America (Fang and Wang 2020). Shade tolerance allows the Norway maple to have better understory performance (Webster et al. 2005) because, compared to other *Acer* species, this species can assimilate carbon and use water and nutrients more efficiently (Kloeppel and Abrams 1995). Sycamore seedlings are most successfully established under conditions with high levels of light, while they are less successful in areas of deep shade (Suszka et al. 1996). On the other hand, sycamores display a broad range of plasticity to light and can become a successful invader as they grow faster in shaded conditions, compared to other species, due to their more efficient photosynthesis (Shouman et al. 2017). Norway maple starts to flower three weeks earlier than sycamore, but morphogenesis of embryos starts earlier in sycamore. As a result, seeds of both species mature and are shed at the same time in autumn (Pukacka 1998). Seeds of both *Acer* species are dormant and completion of germination takes 12–20 weeks for Norway maple and 8–15 weeks for sycamore (Suszka et al. 1996).

Plant growth and development are controlled by redox reactions; more precisely, reactive oxygen species (ROS) initiate redox signals for plant organ morphogenesis based on the transitions between the ascorbate (Asc), glutathione and nicotinamide adenine dinucleotide (NAD) phosphate (NADP) redox couples (Considine and Foyer 2014; Huang et al. 2019). Norway maple (orthodox) and sycamore (recalcitrant) seeds, which are physiologically contrasted in terms of their desiccation tolerance, display contrasting NAD(P) contents and redox states during the seed development (Stolarska et al. 2020), desiccation (Alipour et al. 2020) and particularly at the germination stage, the two species exhibited different redox strategies, which was related to the accumulation of ascorbate in sycamore, and accumulation of glutathione and higher levels of pyridine in Norway maple nucleotides (Alipour et al. 2021). Both NAD and NADP (NAD(P)) are important signaling molecules in plants and function in catabolic and anabolic reactions, respectively, thus orchestrating cellular redox homeostasis (Gakière et al. 2018; Hunt et al. 2004; Mahalingam et al. 2007; Pétriacq et al. 2013). The abovementioned redox couples function as managers to keep redox balance and to maintain metabolic homeostasis (Hasanuzzaman et al. 2019) which are required for organ developmental processes of plants (Arrigoni and De Tullio 2002). They modulate both sensing and transmission of ROS signals thereby affecting cell division, cell differentiation, and cell fate (Foyer and Noctor 2011). Redox homeostasis is essential for cell survival and is regulated at the growth stage via the interplay between NAD(P) pools and the ascorbate and glutathione rendering NAD(P)-based buffering system (Noctor 2006; Pellny et al. 2009).

Photosynthesis is the fundamental process that fuels early seedling growth and determines further plant development contributing to increased fitness of some species. A larger seedling height benefits seedling survival and further vegetation structure and dynamics (Bianchi et al. 2019). Photosynthesis and photorespiration are linked to the content and redox states of NAD(P) (Lim et al. 2020). More precisely, photosynthesis increases stromal NADPH and NADH/NAD^+^ ratio, whereas photorespiration increases the availability of NADH. Recently, NADP^+^ was found to be important for the biogenesis of photosystem I (PSI) (Ji et al. 2022). Infrared gas spectrometry (Douthe et al. 2018) and fluorescence-based approaches (Murchie and Lawson 2013) are powerful strategies in use for evaluating photosynthesis and plant productivity. In situ examination of photosynthesis is determined from concentration-based indices (Sims et al. 2006), including the chlorophyll content index (CCI), which express the ratio of transmittance at 931 nm to 653 nm and can be converted to chlorophyll concentration (CC) (Parry et al. 2014). The CCI is used to predict the aboveground biomass and productivity in crops (Liu et al. 2019). Chlorophyll content is also an indicator of carbon uptake in forest ecosystems (Croft et al. 2015) and is a proxy for leaf photosynthetic capacity (Croft et al. 2017). The whole vegetation can be estimated based on the normalized difference vegetation index (NDVI). Among the many vigor indices, NDVI is a spectroradiometric index reflecting the state of health or vigor of plant growth in field and isolated plants and displays a high correlation with the total plant biomass (Cabrera-Bosquet et al. 2011). NDVI quantifies leaf greenness and is used for seedling growth monitoring and to assess plant responses to stress before visual symptoms appear, as demonstrated for salt-stressed Arabidopsis seedlings (Beisel et al. 2018). Maximum daily photosynthetic rates are positively correlated with NDVI in plants in which canopy development and photosynthetic activity are in synchrony (Gamon et al. 1995).

We intended to determine the redox basis of seedling establishment in two *Acer* species, the Norway maple and sycamore. The content and redox status of the NAD(P), redox couples of ascorbate and glutathione in leaves and roots were integrated with biometric parameters and vegetation indices to specify tissue- or species-dependent variations in seedling from two *Acer* species at standardized conditions.

## Material and methods

### Seedling emergence

Mature Norway maple (*A. platanoides*) and sycamore (*A. pseudoplatanus*) seeds were collected in 2019 from individual trees growing in Kórnik Arboretum (Western Poland, 2°24′37′′N, 17°09′515′′E) in podzolic soil with atmospheric humidity regime type, pH 6.2–7.7 corresponding to 20–150 cm of depth (Kowalkowski and Prusienkiewicz 1959). The seeds were prepared for storage by drying to a 10% water content (WC) for Norway maple and a 30% WC for sycamore and used for the germination test. To start germination, the seeds were imbibed for 24 h, placed on wet paper towels in closed plastic boxes and kept at 3 °C (cold stratification). The seeds with embryonic axes protruding to 5 mm outside the seed coat were considered germinated. The germination speed index (GSI), was calculated according to Maguire (Maguire 1962). The germinated seeds were sown vertically in plastic boxes covered with a lid in three repetitions of 30 seeds in a substrate (1:1, v/v) consisting of quartz sand (< 1 mm fraction) and sieved peat (pH 5.5–6.5). The boxes were kept at 20 °C and a 16/8 h photoperiod under a light intensity of 60 µmol m^–2^ s^–1^ that was provided by a fluorescent lamp (Fluora, Osram™, Germany). The three-month-old seedlings containing four true leaves each were analyzed. Leaves were cut from all seedlings, mixed and used for preparation of samples consisting of 10 randomly selected leaves. Similarly, complete root systems were cut from all seedlings, mixed and used for preparation of samples consisting of three randomly selected root systems.

### Seedling characteristics

WC was measured by drying three leaf samples and roots at 105 °C for 24 h. The fresh leaves and roots were weighed and frozen at –80 °C for later analysis. The chlorophyll content index (CCI) was measured using a CCM-200 plus chlorophyll content meter (Opti-Sciences, USA). The chlorophyll concentration (CC) was calculated using an equation that referred to the *Acer* species (Parry et al. 2014) and was expressed in µmol m^−2^. The NDVI was measured using PlantPen NDVI 310 and expressed as values in the 0–1 range. The NDVI values between -1 and 0 indicate dead plants. The NDVI values range between 0 to 1 for live plants: 0–0.33 for unhealthy plants and sparse vegetation, 0.33–0.66 for moderately healthy plants, and 0.66–1 for very healthy plants (2019).

### Determination of the content of redox couples

To determine the ascorbate pool, the glutathione pool and NAD(P) content, the extract was prepared according to the method described by Queval and Noctor (Queval and Noctor 2007). The leaf (0.5–0.6 g) and root (0.6–0.9 g) samples were ground in 0.2 M HCl or 0.2 M NaOH. The homogenates were centrifuged for 10 min at 4 °C and 14,000 rpm. The extract obtained for Asc and glutathione determination was adjusted to pH 4.5–5. The supernatant obtained for NAD(P) determination was incubated for 2 min at 100 °C, and after cooling, the pH of the samples was adjusted to 6–7. The below described reaction results were measured using an Infinite M200 PRO (TECAN, Männedorf, Switzerland) plate reader and Magellan software.

### Ascorbate determination

The combination of methods described by Hewitt and Dickes (Hewitt and Dickes 1961) and Queval and Noctor (Queval and Noctor 2007) that were successfully applied in *Acer* seeds (Alipour et al. 2021) were used for the ascorbate determination. Total ascorbate (Asc = AsA + DHA) was measured by reducing the extract with 25 mM dithiothreitol at pH 4.7. AsA was analyzed in neutralized extracts by measuring its absorption at 265 nm in a slightly acidic environment. The absorbance measurements were performed in a 0.1 mM acetic acetate buffer containing 5 mM ethylenediaminetetraacetic acid (EDTA). The determination of DHA was calculated by subtracting free AsA from the total Asc.

### Determination of glutathione

The neutralized extract was treated with 2-vinylpyridine (2-VP) for 30 min at room temperature (RT) and centrifuged twice for 15 min at 4 °C and 14,000 rpm. The reaction mixture contained 120 mM NaH_2_PO_4_/10 mM EDTA pH 7.5, 12 mM 5.5′-dithiobis(2-nitrobenzoic) acid (DTNB), 10 mM NADPH, MQ water and extract (to measure total glutathione, GSH + GSSG) or 2-VP-treated extract (to determine the oxidized form GSSG), and glutathione reductase (0.2 U). The measurements were performed at 412 nm. The calculations were based on calibration curves prepared using GSH and GSSG (Sigma-Aldrich, St. Louis, MO, USA) as standards. The redox potential of glutathione (*E*_GSSG/2GSH_) was calculated using the Nernst equation: *E*_GSSG/2GSH_ = *E*_0_ –(RT/nF)log([red]/[ox]). *E*_0_ = –240 mV (at pH 7); R, gas constant (8.314 JK^–1^ mol^–1^); T, temperature [K]; n, number of electrons involved in the reaction; F, Faraday constant (9.6485 104C mol^–1^); red, molar concentration of GSH; ox, molar concentration of GSSG. *E*_0_ was adjusted to *E*_pH_ as described in Schafer and Buettner (Schafer and Buettner 2001).

### Determination of NAD(P)H

The reaction mixture contained 10 mM HEPES/2 mM EDTA (pH 7.5), 1.2 mM 2,6-dichlorophenolindophenol, 10 mM phenazine methosulfate, and neutralized extracts. Glucose-6-phosphate dehydrogenase and 10 mM glucose-6-phosphate were added to measure NADP, and alcohol dehydrogenase and ethanol were added for the measurement of NAD. The kinetic measurements were performed at 600 nm. The levels of the reduced and oxidized forms of NAD(P) were calculated from calibration curves prepared using NADPH, NADP^+^, NADH and NAD^+^ (Sigma-Aldrich) as standards. NAD(P)-originated physiological indices including anabolic redox charge (ARC) and catabolic redox charge (CRC) were calculated using the equation ARC = NADPH/(NADPH + NADP^+^), CRC = NADH/(NADH + NAD^+^) (Lorenc-Plucińska and Karolewski 1994). Phosphorylation capacity of NADKs was calculated as product to substrate ratio NADP^+^/NAD^+^ (NADK1 and NADK2) and NADPH/NADH (NADK3).

### Reduction capacity and activity of NAD(P)H-dependent enzymes

Proteins were extracted from the leaves and roots of Norway maple and sycamore seedlings. The samples were ground in liquid nitrogen in a chilled mortar and pestle and were homogenized in 50 mM K-phosphate buffer (pH 7.0) with 2% polyvinylpyrrolidone. The homogenates were incubated at 4 °C for 1 h with shaking every 15 min and were centrifuged twice at 4 °C and 20,000 rpm for 20 min to obtain a clear supernatant. The protein concentration was measured according to the Bradford method (Bradford 1976).

The measurements were based on the reduction of DTNB to 5-thio-2-nitrobenzoic acid, which produces a strong yellow color that is measured at 412 nm. The reaction mixture contained a reaction buffer consisting of a 50 mM phosphate buffer pH 7.0, 50 mM KCl, 1 mM EDTA, 1 mM DTNB, and protein extract. This reaction enabled the total sulfhydryl thiol species termed reduction capacity to be determined in our study. Determination of the NAD(P)H-dependent reductases activity was measured after the addition of 8 mM NAD(P)H to the reaction mixture. The reactions were measured for 3 min.

### Statistical analysis

The experiment was conducted once. Data are the means of three independent biological replicates ± the standard deviation (STD). Statistically significant differences are indicated with different letters (one-way analysis of variance (ANOVA), followed by Tukey’s test at *p* ≤ 0.05). Proportional data were arcsine transformed prior to analysis. The relationships between particular parameters were examined using Pearson’s correlation coefficient analysis and further principal component analysis (PCA) implemented in JMPpro16 software. R statistical software was used to calculate Pearson’s correlation coefficients separately for the two *Acer* species (R Core Team 2018). The corrplot package was used to construct correlation matrices (Wei and Simko 2017).

## Results

### Germination capacity and seedling establishment

**Fig. 1:**
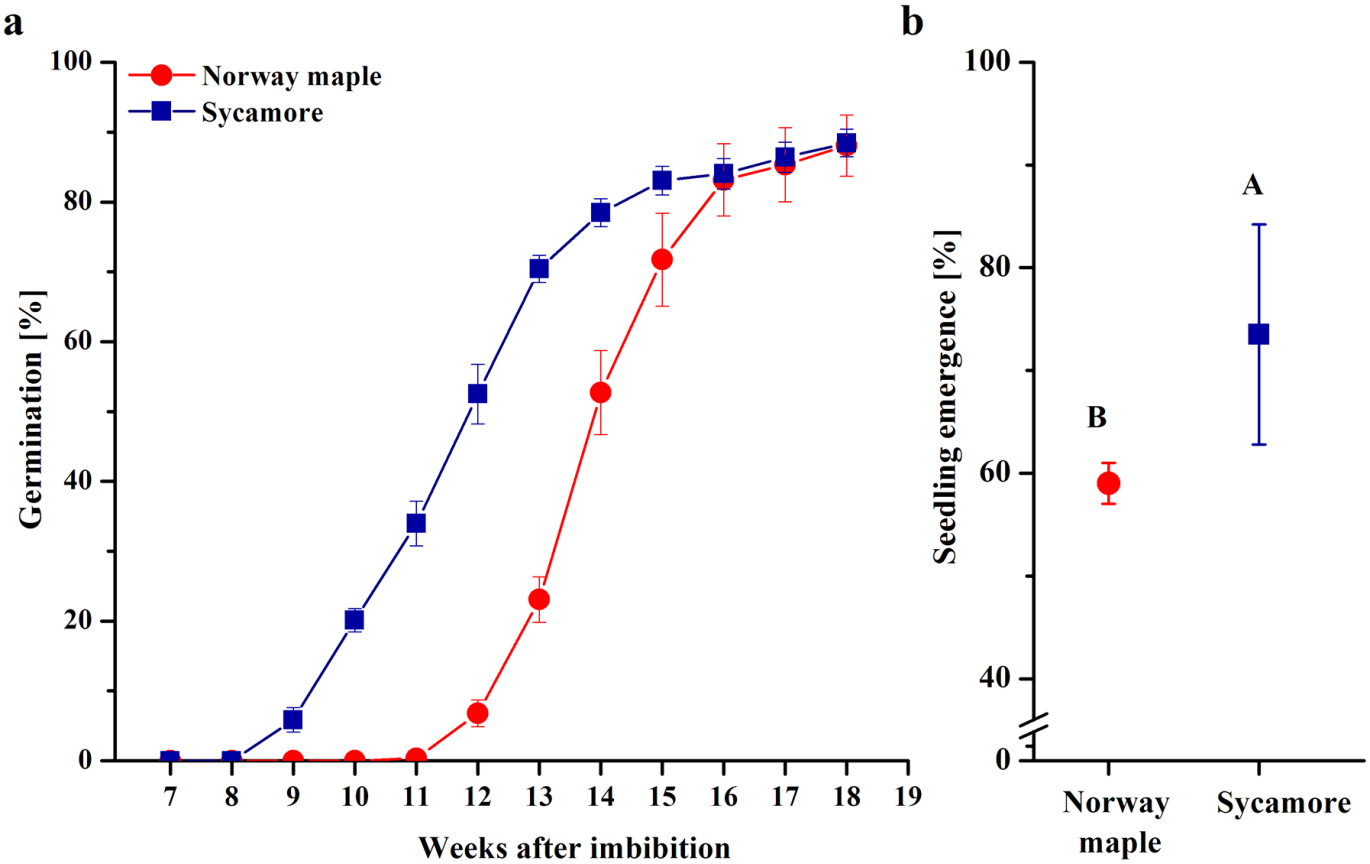
Germination rates of Norway maple and sycamore seeds (**a**) and seedling emergence (**b**) in the two species. Data are the means of three to eight independent replicates ± the standard error

Mature, non stored seeds of Norway maple and sycamore, which were classified as orthodox (resistant to desiccation) and recalcitrant (sensitive to desiccation), respectively, exhibited similar germination capacities, which reached 90%, while the dynamics of germination were different (Fig. 1a). An earlier germination was observed in sycamores than Norway maple seeds. Sycamore seeds exhibited higher germination variability. The GSI, which displays a time-weighted cumulative germination that measures the speed of germination and quantifies seedling vigor, measured during the sigmoidal and linear increase in germination curve in Norway maple and sycamore, respectively, confirmed that the transition to post germination phase was more unified in Norway maple seeds (GSI = 32.4) than in seeds of sycamore (GSI = 26.5).

Germinated seeds were subjected to emergence test. The emergence of seedlings, measured as development of shoot with true leaves, was higher in sycamores than Norway maples, reaching 73.5 ± 12%, and indicated the analyzed replications had a large differentiation (Fig. 1b). The number of established Norway maple seedlings was lower (59.8 ± 2%) but less dispersed in the analyzed replicates.

### Seedling characteristics

The water content of the true leaves of the seedlings was measured. Sycamore leaves contained more water (81.2 ± 0.9%) than that of Norway maple leaves (74.8 ± 0.06%). Similarly, the roots of the sycamore seedlings were more hydrated (88.3 ± 4.7%) than the roots of the Norway maple seedlings (85.4 ± 2%). The seedling structure was compared (Table 1). Sycamore seedlings were higher than Norway maple seedlings; in particular, the epicotyl was longer, whereas the hypocotyl length was comparable in both species. Additionally, the root weight was considerably higher in sycamore seedlings.

**Table 1:** Characteristics of Norway maple and sycamore seedlings

	Shoot (cm)	Hypocotyl (cm)	Epicotyl (cm)	Leaf (g)	Root (g)
Norway maple	7.83 ± 0.17 b	6.36 ± 0.15 a	1.47 ± 0.08 b	0.05 ± 0.0004 a	0.21 ± 0.008 b
Sycamore	9.0 ± 0.17 a	6.0 ± 0.13 a	3.00 ± 0.13 a	0.06 ± 0.001 a	0.34 ± 0.009 a

Means of 60 ± standard error. The same letters indicate groups that are not significantly different according to Tukey’s test

**Fig. 2:**
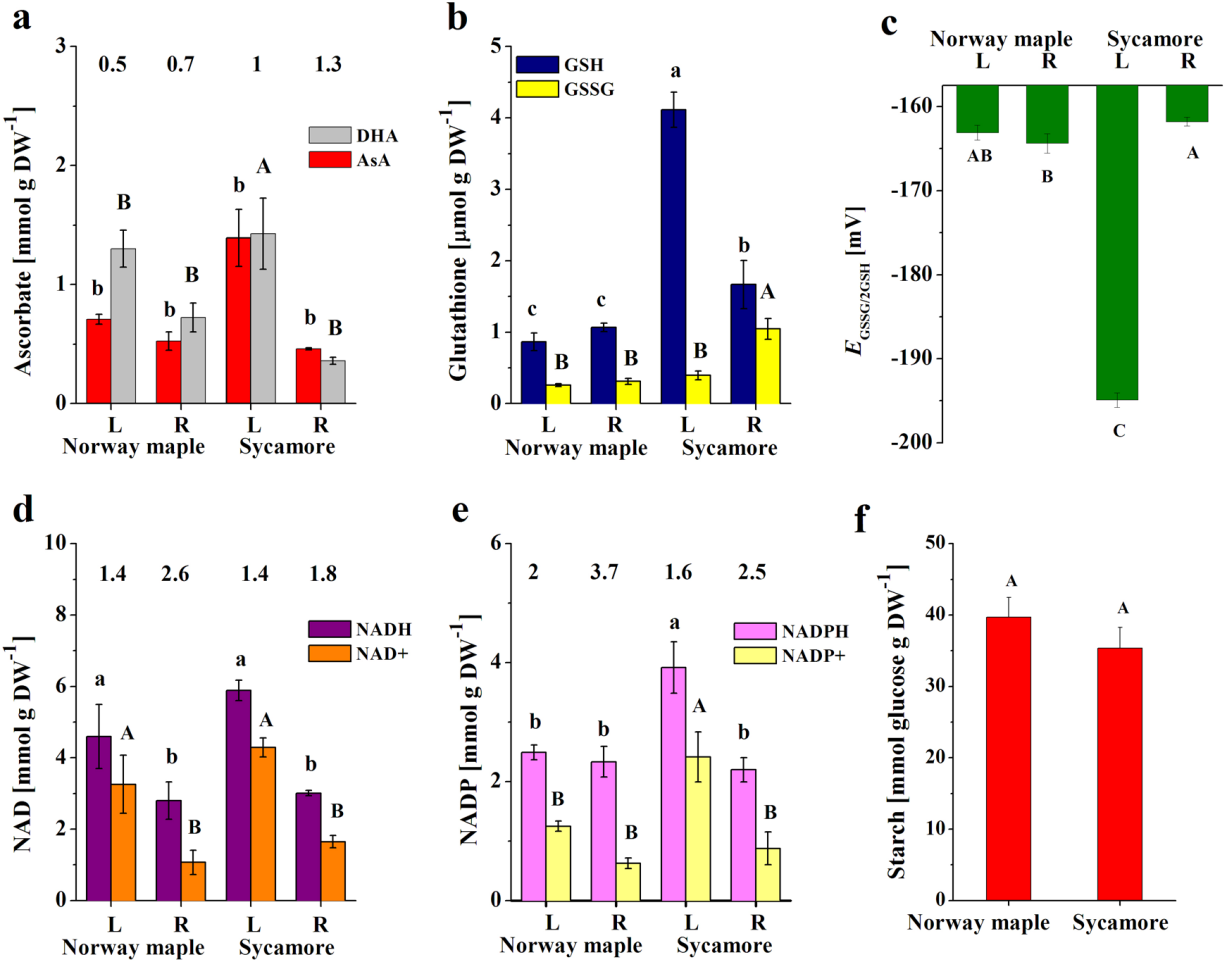
The content of the reduced and oxidized forms of **a** ascorbate (AsA and DHA), **b** glutathione (GSH and GSSG) supported with **c** half-cell reduction potential of glutathione (*E*_GSSG/2GSH_), **d** nicotinamide adenine dinucleotide (NADH and NAD^+^), and **e** nicotinamide adenine dinucleotide phosphate (NADPH and NADP^+^) in the leaves and roots of Norway maple and sycamore seedlings. Starch content was compared in leaves of Norway maple and sycamore seedlings (**f**). The ratio of the content of the reduced to the oxidized form of redox couples is given at the top of a graph. Data are the means of three independent replicates ± the standard deviation

Seedlings that had four true developed leaves were analyzed in terms of NDVI, CCI and CC. Live green vegetation was expressed via NDVI and was significantly higher in sycamore seedlings (Table 2) placing them in the category of very healthy plants. In contrast, the CCI, which is assumed to be an indicator of photosynthetic activity, was higher in Norway maple seedlings than sycamores, which was similar to the calculated CC. The NDVI is used to collect information about the intensity of photosynthesis and to forecast yields or the amount of biomass produced by plants. Despite the lower CC and CCI, the NDVI for the sycamore seedlings was higher than that for Norway maple seedlings (Table 2) emphasizing the evident link between NDVI and seedling biomass, and less compelling with photosynthesis because the efficiency of photosynthesis measured as the content of starch was only slightly higher in Norway maple seeds (Fig. 2f).

**Table 2:** Leaf parameters in Norway maple and sycamore seedlings

	NDVI	CCI	CC (µmol m^−2^)	Starch (nmol glucose g DW^–1^)
Norway maple	0.66 ± 0.009 b	8.84 ± 0.44 a	234.4 ± 9.4 a	39.7 ± 2.7 a
Sycamore	0.77 ± 0.004 a	7.87 ± 0.27 b	214.9 ± 5.9 b	35.3 ± 2.8 a

Means of 60 ± standard error. The same letters indicate groups that are not significantly different according to Tukey’s test

### Redox status

The Asc levels in *Acer* seedlings were higher in leaves than in roots (Fig. 2a). DHA, the oxidized form, was predominant in Norway maple seedlings. However, the redox Asc forms were balanced in sycamore leaves (AsA/DHA ratio = 1), and the reduced form, AsA, was predominant in sycamore roots (AsA/DHA ratio = 1.3). The Asc levels were tripled in sycamore leaves compared with roots, while Norway maple seedlings only differed in DHA content, which was higher in leaves than in roots. AsA/DHA ratio was higher in roots than in leaves, regardless of species. Additionally, AsA/DHA ratio was considerably higher in sycamore seedlings.

Sycamore seedlings exhibited significantly higher total glutathione pool (Fig. 2b). Sycamore leaves contained five times higher levels of the reduced form (GSH) than that of Norway maple leaves and three times higher levels of the oxidized form of GSH, glutathione disulfide (GSSG), in roots. Sycamore roots and leaves differed significantly in the levels of GSSG, whilst Norway maple leaves and roots exhibited equaled GSSG level in the glutathione pool. These observations were reflected in the half-cell reduction potential of glutathione (*E*_GSSG/2GSH_) and highlighted the most reduced cellular environment in sycamore leaves (Fig. 2c).

Similar to Asc, NAD(P) (NAD and NADP) contents were higher in leaves than in roots in sycamore species (Fig. 2d, e). NAD(P) was more abundant in sycamore leaves, whereas the NADH/NAD^+^ ratio was identical in the leaves of both *Acer* species (Fig. 2d). The NAD(P)H content was very similar in the roots and did not differ statistically; however, sycamore roots contained slightly more NAD(P)^+^. As a result, the NAD(P)H/NAD(P)^+^ ratio was the highest in Norway maple roots. In general, NADPH was the predominant form in seedlings, and doubled and tripled levels were reported in Norway maple leaves and roots, respectively, as compared to NADP^+^ (Fig. 2e).

**Fig. 3:**
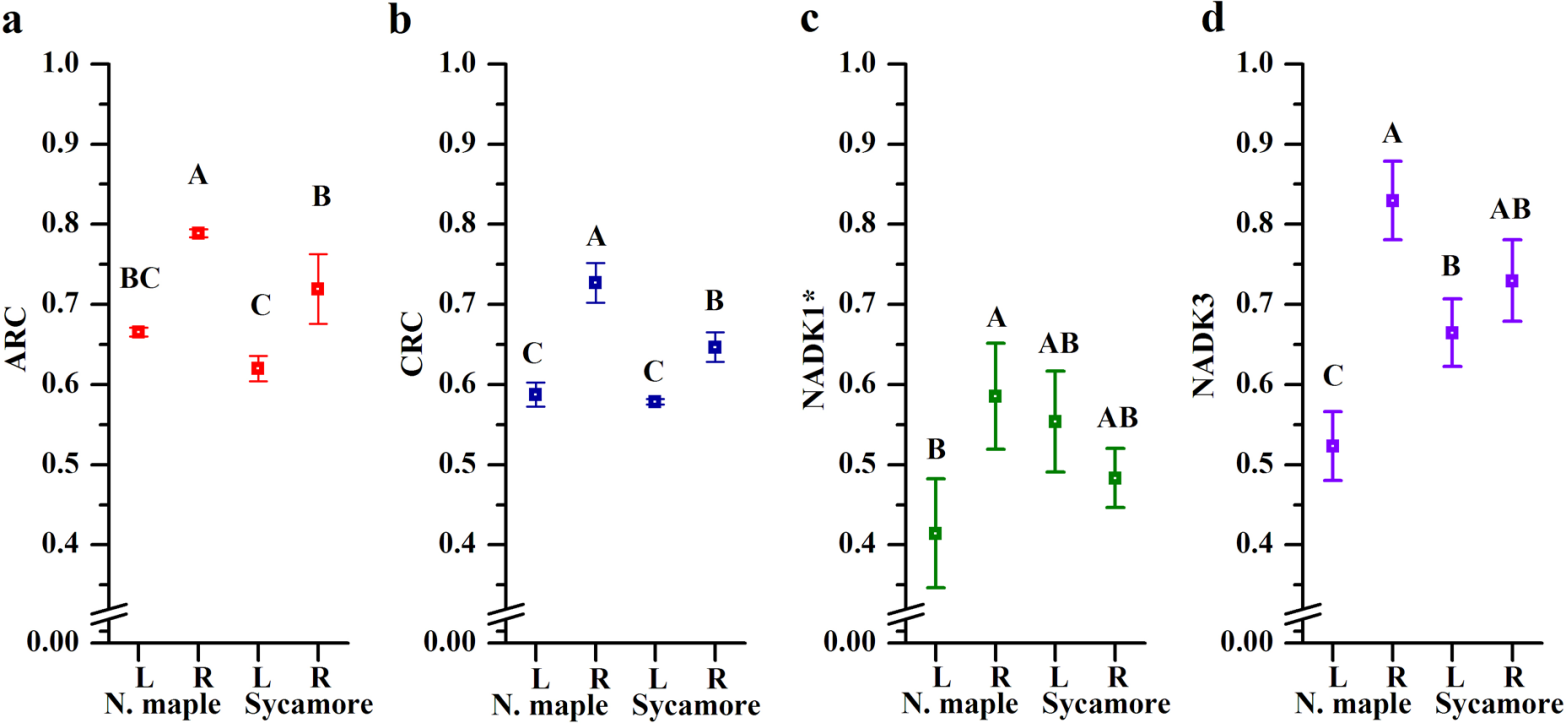
NAD(P)-derived physiological indices **a** anabolic redox charge (ARC), **b** catabolic redox charge (CRC), **c** phosphorylation capacity of cytosolic isoform of NAD kinase (NADK1) and **d** the peroxisomal isoform (NADK3). Data are the means of three independent replicates ± the standard deviation. Asterisk: phosphorylation capacity in leaves is the sum of the activity of NADK1 and chloroplastic NADK2 isoform

Pyridine nucleotides determine anabolic (ARC) and catabolic redox charge (CRC) (Fig. 3 a, b). Both ARC and CRC were equal in leaves of both species. Interestingly, Norway maple roots displayed higher readiness to synthetize and/or decompose than sycamore roots. The efficiency of conversion of NAD to NADP via the activity of NAD kinase (NADK) cytosolic (NADK1), chloroplastic (NADK2) and peroxisomal (NADK1) isoforms was calculated (Fig. 3c, d). Phosphorylation capacity was higher in roots than in leaves of Norway maple seedlings, whereas in sycamore this activity was more unified in whole seedlings.

**Fig. 4:**
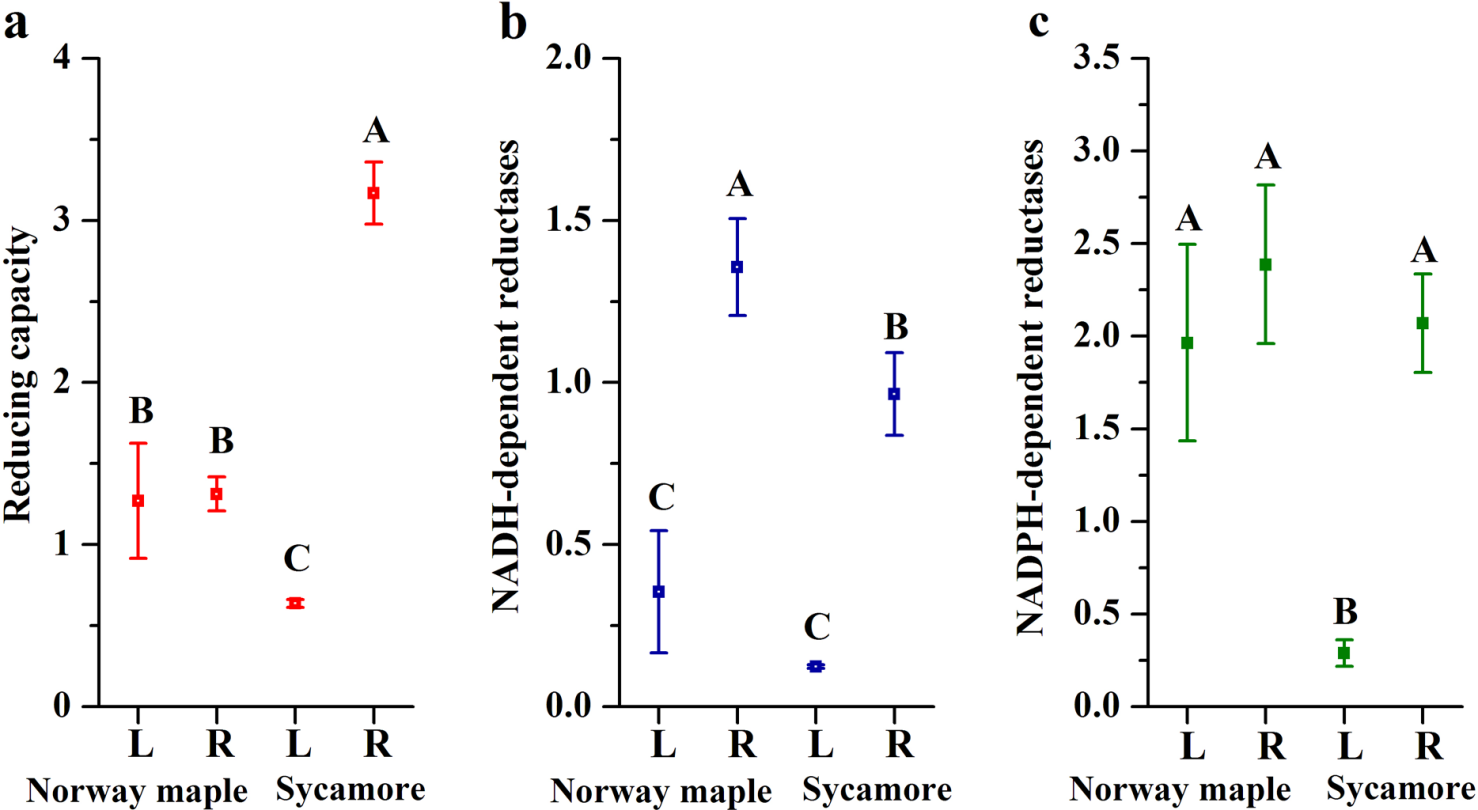
Total reducing capacity (**a**) and the activity of (**b**) NADH-dependent reductases and **c** NADPH-dependent reductases in leaves and roots of Norway maple and sycamore seedlings. Data are the means of three independent replicates ± the standard deviation

There were no differences in the total reducing capacity of compounds containing sulfhydryl groups between leaves and roots of Norway maple seedlings, whereas sycamore roots exhibited five times higher reducing capacity than sycamore leaves (Fig. 4a). Comparing the leaves, the reduction capacity was twice as high in Norway maple seedlings than in sycamore seedlings. The activity of NADH-dependent reductases was eight times higher in the roots than in the leaves of sycamore seedlings, whereas this activity was four times higher in the roots than in the leaves of Norway maple seedlings, and higher activities were found in Norway maple (Fig. 4b). NADPH-dependent reductases exhibited unified activity in tissues and species except for sycamore leaves, in which the activity was markedly lower with 7 times lower values (Fig. 4c).

**Fig. 5:**
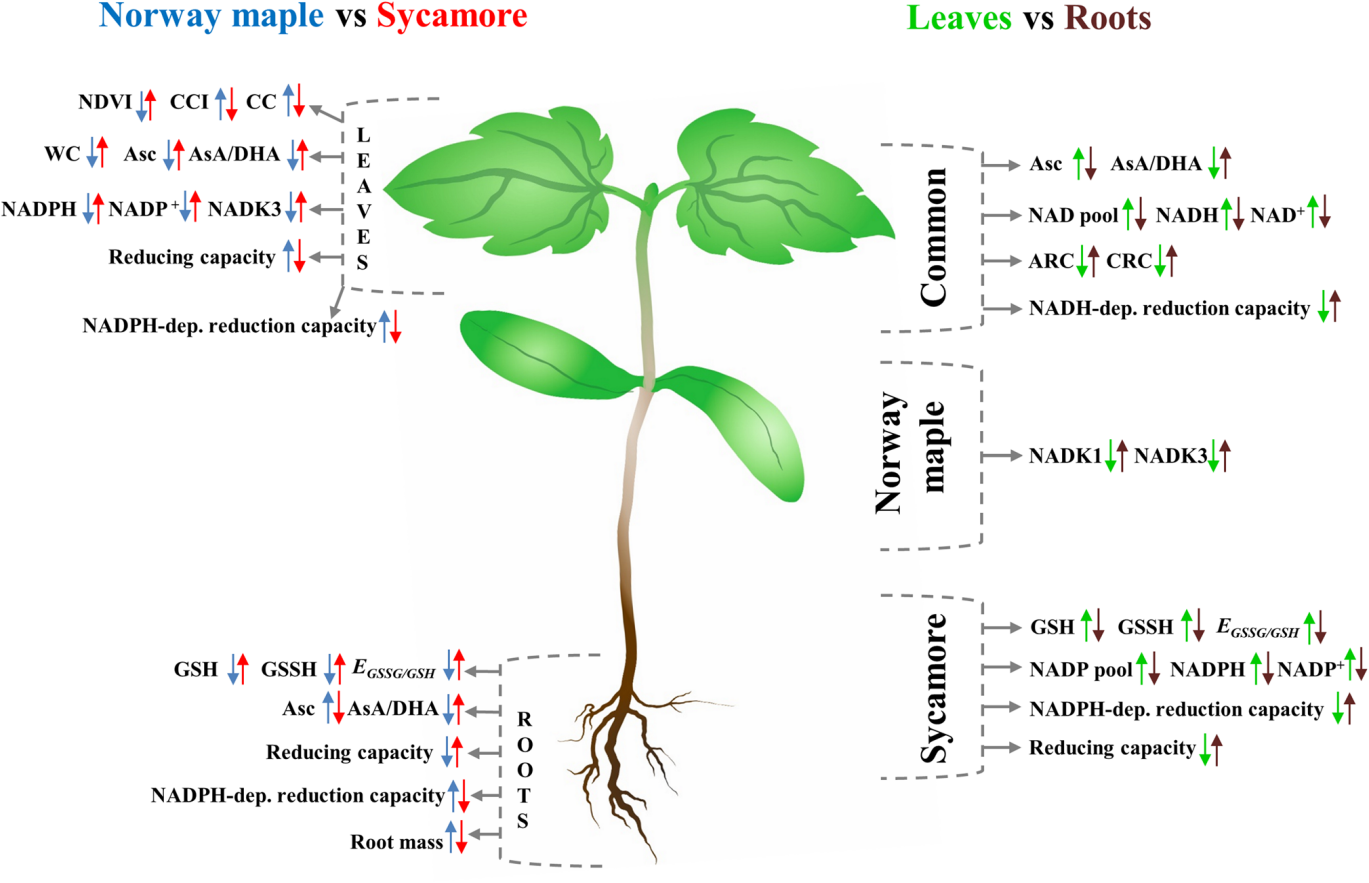
Scheme summarizing the most contrasting characteristics of Norway maple and sycamore seedlings reported in their leaves and roots. Arrow up refers to an increase, arrow down refers to a decrease. Arrow color indicates significant changes reported in Norway maple (blue arrows) as compared to sycamore (red arrows), and in leaves (green arrows) as compared to roots (black arrows)

**Fig. 6:**
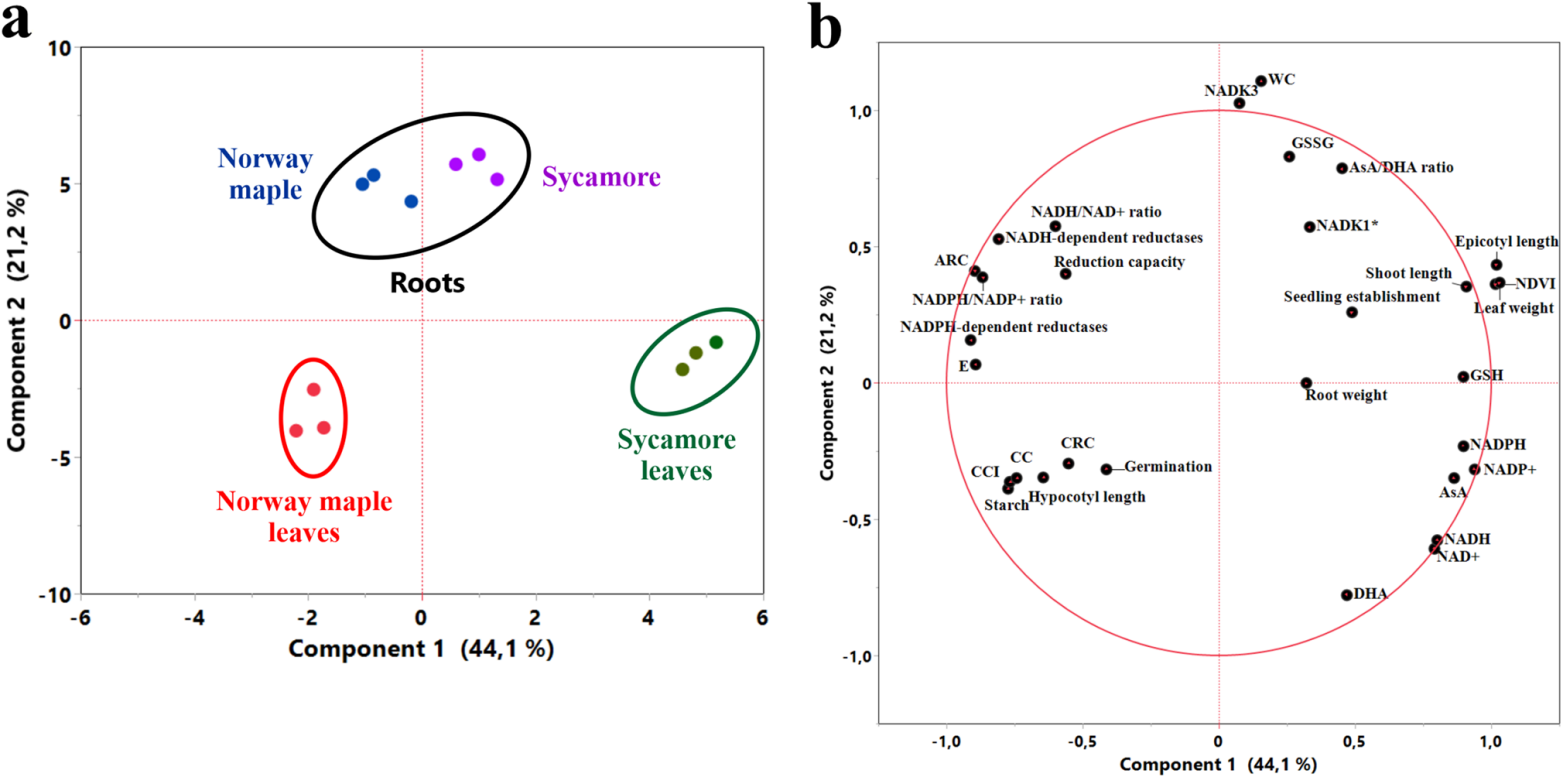
PCA ordination diagram of sample plots (**a**) and quantified variables (**b**). The percentages of explained data variance for each PC are shown on the x and y axes. Reduced (GSH) and oxidized (GSSG) forms of glutathione; reduced (AsA) to oxidized (DHA) forms of ascorbate; reduced (NADH) and oxidized (NAD^+^) forms of nicotinamide dinucleotide (NAD) and their corresponding phosphorylated forms (NADPH, NADP^+^); normalized difference vegetation index (NDVI), chlorophyll content index (CCI), chlorophyll concentration (CC), water content (WC), phosphorylation capacity (NADK1*, NADK3), asterisk: phosphorylation capacity in leaves is the sum of the activity of NADK1 and chloroplastic NADK2 isoform

*Acer* seedling characteristics are summarized and contrasted in Fig. 5 and were further analyzed statistically. PCA analysis (Fig. 6) revealed topscoring variables for PC1 (NDVI, contents of both redox forms of NADP and GSH, epicotyl length and activity of NADPH-dependent reductases) and for PC2 (WC, activity of NADK3, contents of GSSG and DHA and AsA/DHA ratio). A strong positive relation was reported between NDVI and the following variables AsA, GSH, GSSG, and all redox forms of NAD(P) indicating that a link exists between vegetation index and variables describing redox status. Similarly, epicotyl length displayed strong interactions with identical variables related to redox status. WC positively affected growth parameters including epicotyl length and leaf weight, whereas phosphorylation capacity (NADK3) of redox/electron carriers displayed a negative effect on NDVI. Interestingly, correlation matrices performed separately for each species revealed that reduction capacity was positively correlated with ARC uniquely in sycamore seedlings, whereas reduction capacity was negatively correlated with germination capacity uniquely in Norway maple seedlings (Fig. S1). Further, *E*_GSSG/2GSH_ was positively correlated with the activity of NAD(P)H-dependent reductases, redox forms of Asc and negatively correlated with the level of all redox forms of NAD(P) and AsA/DHA ratio exclusively in sycamore seedlings. The above correlations were lacking in Norway maple seedlings.

## Discussion

We investigated seedling performance stage in two *Acer* species, producing seeds with contrasting physiology, in a controlled, stress-free environment to identify inter-tissue, inter-species, and tissue x species differences and to provide the basis for further research on the redox regulation of seedling development in natural ecosystems. Seedlings of Norway maple populations were naturally grown across Europe, and after exposure to increased temperature, decreased moisture and combined stresses, these seedlings were reported to grow larger than the seedlings of sycamores originating from the same locations, especially regarding root biomass (Carón et al. 2015). By using controlled optimal conditions in this study, we documented that shoot height and root biomass were higher in sycamore than in Norway maple (Table 1). In general, Norway maple seedlings were assumed to be more successful than sycamores under the challenges of climate change (Carón et al. 2015). At the same time, sycamore regenerates easily and develops rapidly when they were grown on suitable sites and out-competed other woody plant species (Hein et al. 2009). Observed higher shoot height and root biomass (Table 1) is probably the effect of more rapid growth of sycamore seedlings at this particular developmental stage. Plant growth and development are predominantly determined genetically but are incessantly modified by environmental factors. Redox changes, which are both triggered by the environment and are genetically determined, control plant growth and development, adaptation, damage removal and death (Kocsy et al. 2013). Redox changes regulate or initiate many physiological processes via ROS, antioxidants and plant hormones through reprogramming gene expression and the synthesis of proteins and other compounds related to metabolic processes (Considine and Foyer 2014; Kocsy et al. 2013). Redox changes comprise many thiol-based mechanisms, including the conversion of GSH to GSSG (Rouhier et al. 2015). Mutants lacking genes of the GSH biosynthesis pathway are lethal at the embryo or seedling stage (Cairns et al. 2006; Pasternak et al. 2008). The pool of glutathione (GSH + GSSG) was considerably higher in sycamore seedlings than Norway maple, as it was up to four times higher in leaves and two times higher in roots (Fig. 2b). Glutathione is necessary for shoot apical and root apical meristems to function properly (Rouhier et al. 2015). Therefore, a higher glutathione content potentially contributed to a larger root system and the longer shoots of sycamore seedlings (Table 1), particularly when levels of GSH oppositely affected anabolic reactions in *Acer* seedlings (Fig. S1).

Glutathione combined with ascorbate displays antioxidant functions and redox regulation of signaling pathways via the ascorbate–glutathione cycle, which is termed the Foyer-Halliwell-Asada pathway (Foyer and Noctor 2011). Ascorbate that is accumulated in chloroplasts is essential in plant survival (Zechmann 2018). The ascorbate levels were higher in embryonic axes but lower in the cotyledons of germinated sycamore seeds as compared to Norway maple (Alipour et al. 2021). Sycamore leaves displayed the highest ascorbate content as compared to its roots and whole Norway maple seedlings (Fig. 2a), contributing to better seedling performance in this species. Ascorbate recycling is found in plants and is a crucial element for their adaptation to changes in the environment (Valero et al. 2016), especially when environmental stress elevates the formation of ROS (Bilska et al. 2019). At chloroplastic PSI, oxygen is first reduced to superoxide anion (O_2_^**.**−^) and then further to hydrogen peroxide (H_2_O_2_), and singlet oxygen (^1^O_2_) is produced at PSII. The photosynthetic apparatus is well protected against ROS. H_2_O_2_ is removed via the ascorbate–glutathione cycle (Foyer and Noctor 2011) but the water-water cycle suppresses the formation of ^1^O_2_ (Asada 2006). Higher pools of ascorbate and glutathione, which are predominantly in their reduced forms together with the elevated pools of the reduced NAD(P) forms reported in sycamore leaves (Fig. 2), strongly suggest that the ascorbate–glutathione cycle operates more efficiently in the seedlings of this species principally in leaves displaying the most reduced cellular environment expressed as *E*_GSSG/2GSH_ (Fig. 2c). As a result, sycamore leaves might display improved health indices, including NDVI, compared to that of Norway maple leaves (Table 2).

Air temperature is the main factor shaping the chlorophyll content in *Acer* species (Volodarets et al. 2020). The temperature was equal for both species throughout the experiment, which excluded the temperature factor in our study. A single drought can decrease the sum of chlorophyll in sycamore leaves by over 57%, while in Norway maple, the decrease is up to 22% (Volodarets et al. 2020). We provided identical irrigation for *Acer* seedlings; therefore, the lower CCI in sycamore leaves must have had a different cause. Interestingly, CCI was negatively correlated with the reduction capacity, which was diversified in sycamore leaves and roots and was enormously high in the latter (Fig. 6). It is possible that growth investment was linked more to the roots and shoots than to the leaves because longer shoots and larger roots were reported in sycamore seedlings (Table 1). Furthermore, in sycamore, a shorter hypocotyl and the lower activity of NADPH-dependent reductases were positively correlated with seedling establishment (Fig. 6). Hypocotyl growth is modulated via a transcription factor termed the elongator complex (Woloszynska et al. 2018), which acts as a positive regulator of NAD^+^-induced defense responses (An et al. 2016) and a negative regulator of the responses to oxidative stress (Zhou et al. 2009). ROS are signaling molecules involved in regulation of developmental processes including formation of xylary elements in stem (Marzec-Schmidt et al. 2020). ROS concentrations are regulated by a cascade of redox pairs GSH–GSSG, AsA–DHA, and modulated by NADH–NAD^+^ and NADPH–NADP^+^ (Kocsy et al. 2013) involved in regulation of plant development at various growth stages (Ramakrishnan et al. 2022). In this context, the dynamically active ascorbate–glutathione cycle (Fig. 2) might have contributed to the shorter shoots of Norway maple seedlings (Table 1). Additionally, elongator activity is linked to epigenetic control (Woloszynska et al. 2018). Cellular redox status affects enzymes involved in plant epigenetic modifications (Ramakrishnan et al. 2022). DNA methylation in certain genes of the sycamore genome was found to be a regulator of gene expression, particularly of the photosynthetic genes (Ngernprasirtsiri et al. 1988), suggesting that this feature can also affect photosynthesis rates in this species and needs further investigation.

The NDVI index is used to collect information about the intensity of photosynthesis and to forecast yields or the amount of biomass produced by plants. NDVI led us to categorize sycamore seedlings as very healthy plants and Norway maple seedlings as moderately healthy plants. Higher NDVI corresponded with the larger roots and longer shoots in sycamore (Tables 1, 2). The NAD redox status alters photosynthesis (Dutilleul et al. 2003), and the NADPH/NADP^+^ ratio is slightly higher than 1.0, but the lower values have similar efficiency in sustaining photosynthesis (Lendzian and Bassham 1976). We reported NADPH/NADP^+^ ratios that reached 1.6 in sycamore and 2 in Norway maple leaves (Fig. 2e). The abundance of NADPH possibly affected the rates of photosynthesis in Norway maple leaves, especially when higher CCI values were detected in this species (Table 2) together with slightly higher starch content (Fig. 2f). Probably, the reduction capacity of sulfhydryl compounds together with the activity of NADPH-dependent reductases modulated the redox state in Norway maple more efficiently than GSH. The CCI was higher in the leaves of Norway maple seedlings than in those of sycamore seedlings (Table 2), which is in line with previous observations (Marosz 2009). Both CCI and NDVI reflect plant biomass, however CCI describes aboveground biomass (Liu et al. 2019) and NDVI characterize total plant biomass (Cabrera-Bosquet et al. 2011). In this context, NDVI in *Acer* seedlings was associated predominantly with root biomass and shoot height.

For normal growth and development, plants must closely control their NAD levels (Wang and Pichersky 2007), and the excess of NADPH is exported from chloroplasts to other cellular compartments (Hashida and Kawai-Yamada 2019). NAD content is a modulator of the whole metabolism (Dutilleul et al. 2005), and the sycamore leaves contained a higher NAD pool (Fig. 2d). Furthermore, the availability of the reduced NAD form stimulates the rate of nitrogen assimilation and carbon metabolism (Dutilleul et al. 2005). The NADH/NAD^+^ ratio was equal in the seedlings of both *Acer* species and higher than 1 (Fig. 2d), emphasizing that NADH was available for energy production and growth processes. Cell proliferation in the shoot apical meristem is under redox control. More precisely, shoot growth is regulated via ROS produced by NADPH oxidases (Schippers et al. 2016). Other growth-related functions of NADPH oxidases include regulation of root development and leaf morphogenesis (Hu et al. 2020). In this context, more abundant NADPH in sycamore seedlings (Fig. 2e) might contribute to longer shoots in this species (Table 1). In contrast, more efficient phosphorylation capacity of NAD reported in Norway maple roots was not reflected in root biomass (Table 1) indicating that the expected output of enzymes does not necessarily correlate with the abundance of their substrates and/or cofactors.

Some oxidative characteristics might be transferred from seeds to seedlings, and seedlings originating from orthodox seeds employ various, species-specific, redox strategies to grow (Wawrzyniak et al. 2020). Both *Acer* species displayed distinct redox control during seed development (Stolarska et al. 2020), seed desiccation (Alipour et al. 2020) and seed germination (Alipour et al. 2021). *Acer* seedlings exhibited NAD(P) redox characteristics (Figs. 2, 3) similar to the one demonstrated in germinated seeds (Alipour et al. 2021). Importantly, major differences in redox status regulation appeared in *Acer* leaves (Fig. 2d, e). Young and mature Norway maple leaves display different redox strategies and photosynthetic performance (Lepeduš et al. 2011). Less data is available for sycamore species but our results might inspire more detailed research on redox biology in this species. Redox regulation adjust photosynthesis (Wormuth et al. 2007) and starch metabolism (Skryhan et al. 2018) emphasizing that seedling establishment is modulated via redox status and more investigations are needed to elucidate the redox basis of survival and growth of trees.

Summarizing, the leaves and roots of the two *Acer* seedlings demonstrate distinct growth parameters and presumably different mechanisms to regulate the redox couple contents and states of the two antioxidants ascorbate and glutathione and the major electron acceptor/donor NAD(P). The higher biomass of the sycamore seedlings was reflected in the higher NDVI and levels of the NADP redox forms that propel the anabolic reactions related to growth. However, the intensity of photosynthesis was likely higher in the leaves of Norway maple seedlings. The global reducing capacity, which was higher in the leaves of Norway maples and in the roots of sycamore, indicates that different parts of *Acer* seedlings are better prepared to quickly neutralize environmental stress signals, resulting in oxidative stress.

## Supplementary Information

Below is the link to the electronic supplementary material.

Supplementary file1 (PDF 414 KB)
