# Supplementary material for: Distinct redox state regulation in the seedling performance of Norway maple and sycamore
Source: J Plant Res. 2022 Nov 17;136(1):83–96. doi: 10.1007/s10265-022-01419-3 (PMC9831958; doi:10.1007/s10265-022-01419-3)

Distinct redox state regulation in the seedling performance of Norway maple and sycamore

Journal of Plant Research

Shirin Alipour[1], Natalia Wojciechowska[1,2], Barbara Bujarska-Borkowska[1], Ewa Marzena Kalemba[1*]

[1] Institute of Dendrology, Polish Academy of Sciences, Kórnik, Poland.

[2] Department of General Botany, Institute of Experimental Biology, Faculty of Biology, Adam Mickiewicz University, Uniwersytetu Poznańskiego 6, Poznań, Poland.

* Corresponding author, mail: kalemba@man.poznan.pl

Figure S1 Correlation matrices. Correlation matrices calculated for Norway maple (a) and sycamore (B) seedlings between germination capacity, seedling emergence, contents of nicotinamide adenine dinucleotide (NAD) and its phosphate (NADP) redox couples (reduced NAD(P)H and oxidized NAD(P)$^+$) and their ratios (NAD(P)H/NAD(P)$^+$), levels of ascorbic acid (AsA), dehydroascorbate (DHA) and their ratio (AsA/DHA), contents of reduced (GSH) and oxidized (GSSG) glutathione, catabolic redox charge (CRC), anabolic redox charge (ARC), phosphorylation capacity of isoform 1 (NADK1) and isoform 3 (NADK3) of NAD kinase, half-cell reduction potential of glutathione ($E_{GSSG/2GSH}$), water content (WC), reduction capacity, and activity of NAD(P)H-dependent reductases. Proportional data were transformed prior to analysis using the arcsine transformation. Empty squares indicate non-significant correlation (P > 0.05). Data available for whole seedlings including leaf and root characteristics was used for construction of correlation matrices.

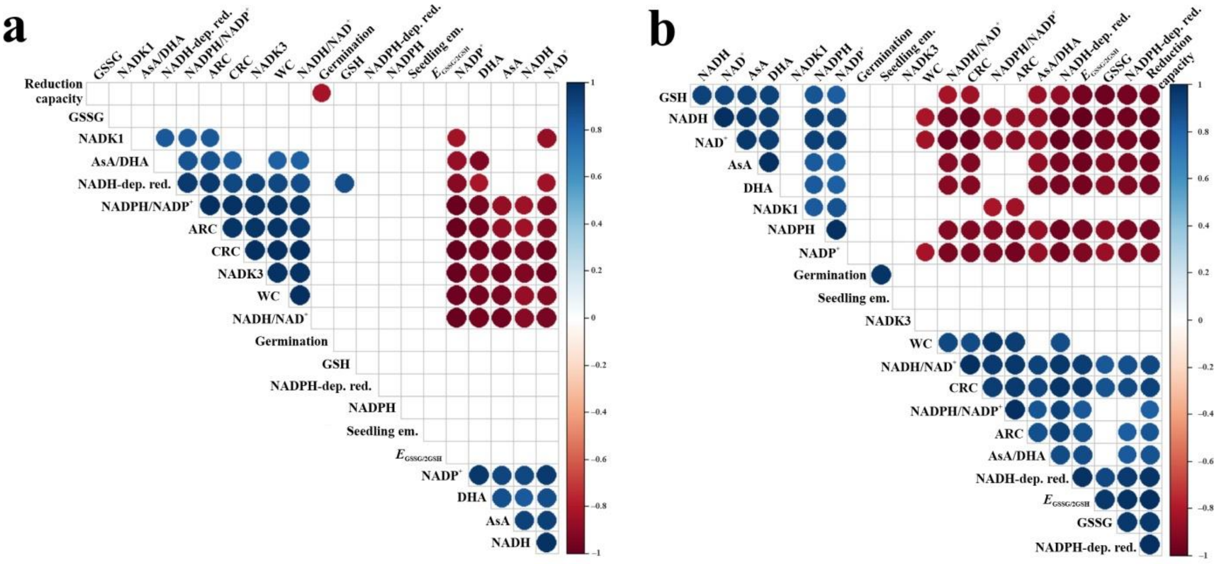

Supplement: Supplementary file 1 — Supplementary file1 (PDF 414 KB) [file 10265_2022_1419_MOESM1_ESM.pdf]
